# Germline variation of Ribonuclease H2 genes in ovarian cancer patients

**DOI:** 10.1186/s13048-020-00753-1

**Published:** 2020-12-22

**Authors:** Rahel Polaczek, Peter Schürmann, Lisa-Marie Speith, Robert Geffers, Matthias Dürst, Peter Hillemanns, Tjoung-Won Park-Simon, Clemens Liebrich, Thilo Dörk

**Affiliations:** 1 Department of Gynaecology and Obstetrics, Gynaecology Research Unit (OE 6411), Hannover Medical School, Carl-Neuberg-Str. 1, D-30625 Hannover, Germany; 2 Genome Analytics Unit, Helmholtz Institute for Infection Research, Braunschweig, Germany; 3 Department of Gynaecology and Obstetrics, University Clinics Jena, Jena, Germany; 4 Department of Gynaecology and Obstetrics, Hospital Wolfsburg, Wolfsburg, Germany

**Keywords:** RNase H2, Ribonucleotide excision repair, Homologous recombination, MMR deficiency, Platinum resistance, Epithelial ovarian carcinoma

## Abstract

Epithelial ovarian carcinoma (EOC) is a genetically heterogeneous disease that is partly driven by molecular defects in mismatch repair (MMR) or homology-directed DNA repair (HDR). Ribonuclease H2 serves to remove mis-incorporated ribonucleotides from DNA which alleviates HDR mechanisms and guides the MMR machinery. Although Ribonuclease H2 has been implicated in cancer, the role of germline variants for ovarian cancer is unknown. In the present case-control study, we sequenced the coding and flanking untranslated regions of the *RNASEH2A*, *RNASEH2B* and *RNASEH2C* genes, encoding all three subunits of Ribonuclease H2, in a total of 602 German patients with EOC and of 940 healthy females from the same population. We identified one patient with a truncating variant in *RNASEH2B*, p.C44X, resulting in a premature stop codon. This patient had high-grade serous EOC with an 8 years survival after platinum/taxane-based therapy. Subsequent analysis of TCGA data similarly showed a significantly longer progression-free survival in ovarian cancer patients with low *RNASEH2B* or *RNASEH2C* expression levels*.* In conclusion, loss-of-function variants in Ribonuclease H2 genes are not common predisposing factors in ovarian cancer but the possibility that they modulate therapeutic platinum response deserves further investigation.

**Supplementary Information:**

The online version contains supplementary material available at 10.1186/s13048-020-00753-1.

## Introduction

Ovarian cancer is a genetically heterogeneous disease [13]. The susceptibility to epithelial ovarian carcinoma can be inherited and then frequently associates with germline variants affecting mismatch repair (MMR) or homology-directed repair (HDR). Loss-of-function variants in the MMR genes *MLH1, MSH2, MSH6* and *PMS2* have been reported as risk factors for clear cell and endometrioid ovarian carcinomas [15, 21]*.* Loss-of-function variants in the HDR genes *BRCA1*, *BRCA2*, *PALB2*, *BRIP1*, *RAD51C* and *RAD51D* have been described, among others, to strongly increase the risk for serous ovarian cancer [19, 21–25]. In addition, evidence has been accumulated that deleterious variants in HDR genes are also predictors of therapeutic outcome [16, 18]. This seems to be related to the nature of therapeutic agents commonly used in ovarian cancer treatment, such as platinum compounds, which cause interstrand crosslinks and double-strand breaks in tumor cell DNA. The repair of this damage is dependent on HDR proficiency.

Human Ribonuclease H2 (RNAse H2) has been identified to interact with both, HDR and MMR pathways. RNase H2 is a trimeric enzyme composed of three subunits encoded by the *RNASEH2A*, *RNASEH2B* and *RNASEH2C* genes [12, 14]. It initiates ribonucleotide excision repair (RER) for the error-free removal of mis-incorporated ribonucleotides within a DNA strand [14]. The impairment of RNase H2 function results in the accumulation of ribonucleotides in genomic DNA. Consecutive chronic low-level DNA damage can then give rise to systemic autoimmunity in heterozygotes [10] and can result in the inflammatory Aicardi-Goutières syndrome with biallelic pathogenic variants [5]. The low-level damage in the absence of RNase H2 appears to result from an alternative TOP1-mediated processing of rNMPs which is a source of PARP1 trapping and 3′-blocking lesions [28]. If not removed properly, HDR-deficient cells are exquisitely sensitive to such lesions [2, 3, 28]. As the ribonucleotide excisions by RNase H2 may also serve to orient the mismatch repair machinery, RNase H2 defects may furthermore decrease the efficiency of MMR [9, 14, 17]. Hence, RNAse H2 impacts on repair pathways that are critical for ovarian cancer development.

Although RNase H2 has been implicated in cancer, there is little data available about germline variants of its three genes in cancer patients, and their role in ovarian cancer risk and prognosis is unclear. In the present study, we have thus sequenced the *RNASEH2A*, *RNASEH2B* and *RNASEH2C* genes in a case-control series of 602 German patients with EOC and of 940 healthy females from the same population.

## Patients and methods

### Patients

Ovarian cancer cases were included from a hospital-based case-control study which included 602 German patients who had been diagnosed with epithelial ovarian adenocarcinoma at Hannover Medical School (*n* = 305), the Wolfsburg Gynecology Clinics (*n* = 135), the University of Jena (*n* = 86), or partner hospitals in Bremen, Braunschweig, Burgwedel, Kassel, Oldenburg and Lüneburg (*n* = 76). A defined histological subtype had been assigned to 505 of the 602 patients (83.9%). The most common histology was serous (*n* = 381, including 276 high grade, 56 low grade, 49 no grade recorded), followed by endometrioid (*n* = 61), mucinous (*n* = 47), clear cell (*n* = 12), and 4 rare histological subtypes. Median age at diagnosis was 61 years, and 3% of patients reported a first-degree family history of ovarian cancer. Informed written consent was obtained from each patient. The control group consisted of 940 samples collected from unrelated healthy female blood donors at Hannover Medical School, Lower Saxony, Germany. Median age at study entry was 30 years (range 18–68 years). 95% were Germans, and all were Europeans, they were cancer-free up to the time of blood draw and were unrelated to the patients. For each study participant, DNA was isolated from peripheral white blood cells using standard phenol-chloroform extraction. The study was approved by the Ethics Commission at Hannover Medical School.

### Molecular analyses

Target-specific primers for resequencing were obtained from Fluidigm Corp (San Francisco) using Fluidigm primer service program. Twenty-five primer pairs were designed and validated to cover the *RNASEH2A*, *RNASEH2B* and *RNASEH2C* exons in partially overlapping fragments of 250–300 bp (Supplementary Table S1). For the multiplex PCR, each genomic DNA sample was normalised to a concentration of ~ 100 ng/μl, as measured by NanoDrop photometry, and loaded onto a microfluidic 48.48 Access Array IFC (Fluidigm, San Francisco). Each primary primer pair contained the template specific sequence and a tag sequence. Each secondary primer pair contained the anti-tag sequence, a sample-specific unique barcode, and the Illumina adaptor sequence. PCR products harvested from each sample were checked through agarose gel electrophoresis to confirm uniformity of the amplicon coverage. The success rate at this stage was 92%. PCR products from cancer samples and control samples were pooled in eight libraries which were purified using AMPure magnetic beads and quantified using Quant-iT PicoGreen dsDNA Assay kit. Each library was sequenced on a separate run on a MiSeq system (Illumina, San Diego, CA). Paired end sequencing was performed using MiSeq Reagent Kit v3 (2 × 300 cycles). Sequencing quality was evident by the Q30 scores (one error in 1000 bp sequence) and a cutoff of 85% sequence with Q30 score was used as an indication of successful sequencing.

The *RNASEH2B* variant p.C44X was additionally amplified from germline DNA of the patient and a control using the primers 5′- ACAGGGTAAAGTAAGGTGAG - 3′ and 5′- GTGTATATACTCATTAGCCAC - 3′ and was validated through Sanger sequencing using BigDye chemistry and capillary electrophoresis on a SeqStudio Genetic Analyser (Applied Biosystems).

### Bioinformatics

Sequencing data were analysed with NextGENe 2nd Generation Sequencing Software v.2.4.2 (SoftGenetics, Philadelphia, USA). Briefly, fastq.qz files were aligned to *RNASEH2A*, *RNASEH2B* and *RNASEH2C* gbk files from the human reference sequences of the GRCh37.p13 Assembly (NC_000019, 12,912,863..12924462 for *RNASEH2A,* NC_000013, 51,483,814..51544596 for *RNASEH2B,* NC_000011 complement (65,485,144..65488409) for *RNASEH2C*; http://www.ncbi.nlm.nih.gov/gene/). Alignment was performed with a required matching of over 80% within more than 30 bases. We filtered out variants if the proportion of variant calls was below 25% or if the total number of variant calls was below *n* = 5. The average number of sequence variants detected in cases was very similar to those detected in controls (cases: median 187 variants (range 98–404); controls: median 192 variants (range 74–247)). The p.C44X variant in *RNASEH2B* was validated by conventional Sanger sequencing using BigDye chemistry. Sequence variants were checked for previously published reports in the Genome Aggregation database (https://gnomad.broadinstitute.org/) and were annotated with Reference SNP cluster IDs from the NCBI SNP database (http://www.ncbi.nlm.nih.gov/snp). Splice site analyses were performed using the publicly available MaxEntScan tool from Yeo and Burge (http://hollywood.mit.edu/burgelab/maxent/) [8]. Missense variants were assessed for deleteriousness using Mutation Taster (http://www.mutationtaster.org/). Somatic variations of *RNASEH2B* in the Pan Cancer cohort of the TCGA database (4742 tumors, including 316 ovarian tumors) were accessed via the TumorPortal (http://www.tumorportal.org/view?geneSymbol=RNASEH2B).

### Statistics

Genetic association analyses of selected variants were performed using Fisher’s exact test at 2 df, and *p*-values above α = 0.05 were considered non-significant. Survival analysis of TCGA data for ovarian cancer patients was performed using an online Kaplan-Meier plotter (KMplotter, http://kmplot.com/analysis/index.php?p=service&cancer=ovar) [11]. We examined gene expression levels using default analysis conditions, with auto-select best cut-off and for all datasets comprising up to 1435 patients. The Affymetrix probes used were 203022_at for *RNASEH2A*, 229210_at for *RNASEH2B* and 227543_at for *RNASEH2C*. Because we analysed three genes in three patient groups (overall, serous histology, high-grade), *p*-values ≤0.005 were considered significant after Bonferroni correction for multiple testing. Hazard ratios are expressed for high-expression relative to low-expression group. We examined *RNASEH2B* expression levels in the Human Protein Atlas database (https://www.proteinatlas.org/ENSG00000136104-RNASEH2B/pathology/ovarian+cancer#ihc) [26] for 373 ovarian cancer patients using default analysis conditions, with auto-select best cut-off (FPKM 3.5). We compared 82 patients with high expression and 291 patients with low expression and derived a log-rank *P* value for the Kaplan-Meier plot of patient survival in these two groups.

## Results

**Fig. 1:**
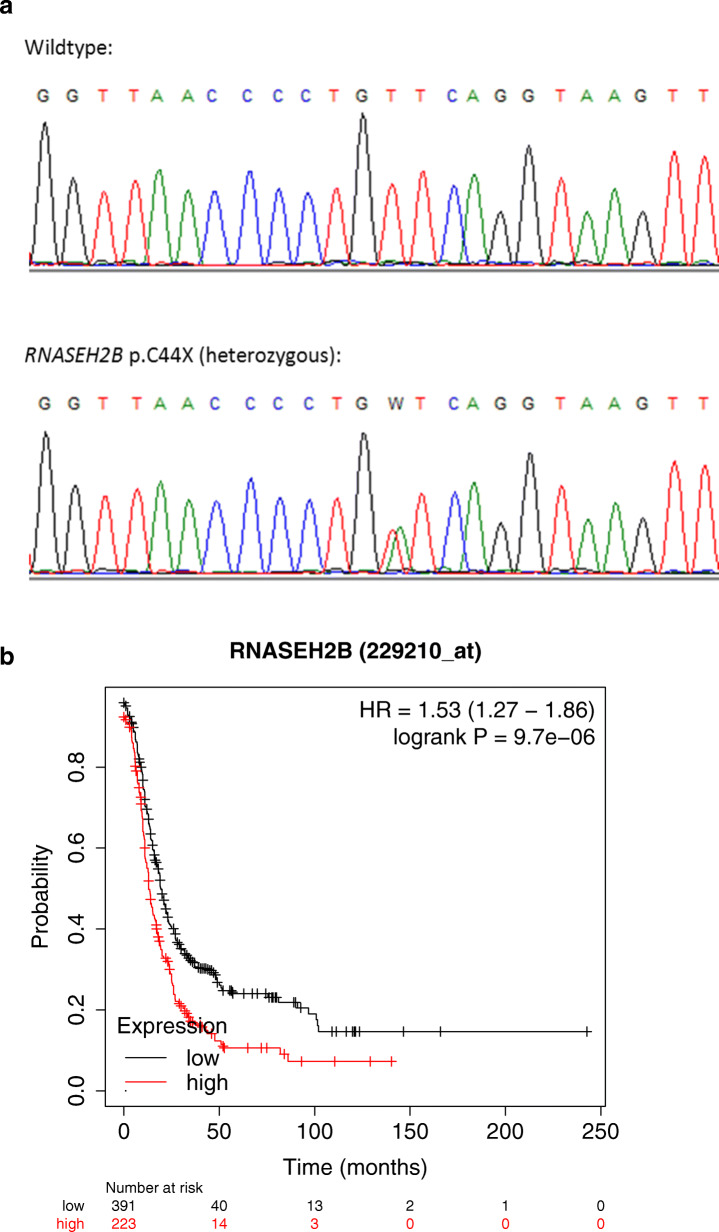
**a**. Sequencing of *RNASEH2B* variant p.C44X. Figure legend: Validation of *RNASEH2B* variant p.C44X through Sanger sequencing in a wildtype control (top) and the index ovarian cancer patient (bottom). The sense strand is shown. Heterozygosity for a T > A transversion was confirmed, changing the cysteine codon TGT to the stop codon TGA. **b**. Progression-free survival of ovarian cancer patients in relation to *RNASEH2B* levels. Figure legend: Progression-free survival of ovarian cancer patients in the TCGA database for patients with high versus low RNA levels of *RNASEH2B*. The Affymetrix probe 229210_at specific for *RNASEH2B* was used, and patients were stratified using auto-select best cut-off in KMplotter (http://kmplot.com/analysis/index.php?p=service&cancer=ovar) [11]

We successfully sequenced the coding exons and their flanking intronic and untranslated regions of the *RNASEH2A*, *RNASEH2B* and *RNASEH2C* genes in a case-control series of 602 German patients with histologically confirmed EOC and of 940 healthy German females. The coding variants identified in this series are summarized in Table 1. In *RNASEH2A*, eight rare missense variants were detected of which three affected conserved residues and were predicted to be pathogenic (p.D2Y/p.L3P, p.G132D, p.R239C). However, these variants were restricted to single females and no overall enrichment was observed in cases compared to controls. In *RNASEH2B*, one truncating variant, p.C44X, was detected in a single ovarian cancer patient (Fig. 1a**)**. Clinical features of this patient will be described further below. We also noted a potential frameshift variant, c.827dupA, in one control. However, the frameshift affects only isoform 1 and is absent/intronic in isoform 2, suggesting that it can be circumvented through means of alternative splicing (Supplementary Fig. S1). Again, no significant overall enrichment was observed for those rare missense variants that affected conserved residues and were predicted to be pathogenic (p.A177T, p.S217P, p.K248N, p.T263A)*.* In *RNASEH2C*, we did not observe any missense or truncating variants*.* One of three synonymous variants, c.468G > T, affected the last base of an exon and was predicted to mildly disturb the splice donor site (MaxEntScan score 7.39 compared to 10.49 for wildtype). However, this variant was not significantly enriched in cases versus controls (OR 1.96, *p* = 0.32).

**Table 1:** Missense and splice site variants in *RNASEH2A*, *RNASEH2B* and *RNASEH2C*

Genome variant	cDNA	Protein	rsID	Predictions	***N*** (cases)	***N*** (controls)
***RNASEH2A***						
chr19:12917491 G > T, chr19:12917495 T > C	c.4G > T, c.8 T > C	p.D2Y, p.L3P	rs761331717, rs764685443	likely pathogenic, variants not conserved	0	1**
chr19:12918043 G > C	c.223G > C	p.E75Q	rs753695101	likely pathogenic, variant conserved	1	0
chr19:12918304 G > A	c.395G > A	p.G132D	rs753110328	likely pathogenic, variant not conserved	1	0
chr19:12921186 T > C	c.605 T > C	p.L202S	rs7247284	likely benign, variant not conserved	41	59 (1 hom)
chr19:12921196 T > A	c.615 T > A	p.D205E***	rs62619782	likely pathogenic, variant conserved	16	16
chr19:12923921 A > G	c.662A > G	p.K221R	rs143534021	likely benign, variant conserved	0	1
chr19:12923974 C > T	c.715C > T	p.R239C	rs372667206	likely pathogenic, variant not conserved	1	0
chr19:12924260 G > A	c.880G > A	p.E294K	rs764614950	likely pathogenic, variant conserved	1	0
***RNASEH2B***						
chr13:51501610 T > A	c.132 T > A	p.C44X	rs74876702	likely pathogenic	1	0
chr13:51517475 A > G	c.455A > G	p.N152S	rs146451037	likely benign, variant conserved	1	1
chr13:51519581 G > A	c.529G > A	p.A177T	rs75184679	likely pathogenic, variant not conserved	0	4
chr13:51522138 A > T	c.632A > T	p.Y211F	rs779596970	likely pathogenic, variant conserved	1	0
chr13:51522155 T > C	c.649 T > C	p.S217P	rs778933609	likely pathogenic, variant not conserved	1	0
chr13:51528043 A > C	c.744A > C*	p.K248N*	rs748144224	likely pathogenic*, variant not conserved	1	0
chr13:51528086 A > G	c.787A > G*	p.T263A*	rs150363383	likely pathogenic*, variant not conserved	1	0
chr13:51530494 insA	c.827dupA*	p.N276Kfs*	rs746868812	likely pathogenic*	0	1
chr13:51530501 G > A	c.830G > A*	p.S277N*	rs200802557	likely pathogenic, variant conserved*	0	1
chr13:51530530 G > T	c.859G > T*	p.A287S*	rs144408326	likely pathogenic, variant conserved*	3	7
chr13:51530539 G > A	c.868G > A*	p.D290N*	rs201190805	likely pathogenic, variant conserved*	0	1
***RNASEH2C***						
chr11:65487516 C > A	c.468G > T	p.A156A	rs61736590	splice site affected	5	4

Missense and splice site variants of *RNASEH2A*, *RNASEH2B* and *RNASEH2C* identified among 602 German patients with EOC and of 940 healthy German females. Variant positions refer to the GRCh37.p13 Primary Assembly of the human genome. Variant annotations are based on reference sequences NM_006397.2, NM_024570.3 and NM_032193.3. RsID was derived from the NCBI SNP database (https://www.ncbi.nlm.nih.gov/snp/). Predictions were made using Mutation Taster and MaxEntScan as described in the Methods section. N (cases) and N (controls) list the numbers of carriers in the respective group. All variant carriers were heterozygotes, except for one homozygote with p.L202S in *RNASEH2A*. Asterisks: * variants that are coding only in isoform 1 of the *RNASEH2B* transcript; **two variants listed separately in NCBI SNP but constituting a double missense allele in the same individual; ***samples with p.D205E also carried p.L202S

We more thoroughly inspected the clinical records of the single patient who was heterozygous for the RNASEH2B*p.C44X truncating variant. This patient had no family history of cancer and had been negative for pathogenic variants after sequencing the *BRCA1*, *BRCA2*, *BRIP1*, *PALB2*, *RAD51C*, *RAD51D*, *PTEN* and MMR genes. She had been diagnosed by the age of 67 years with a high-grade serous ovarian carcinoma of FIGO stage IIIc, pT3c, and with positive nodal status though no detectable metastases. She received standard therapy of six cycles carboplatinum/taxol. Six years later, she presented with a late recurrence and with metastases in breast and abdominal wall. Her cancer then progressed despite nine cycles carboplatinum monotherapy followed by six cycles of treosulfan, three cycles of Caelyx and finally tamoxifen therapy. The patient died from her cancer by the age of 75, eight years after the initial diagnosis. The long interval between her primary diagnosis and the recurrence was markedly higher than the median progression-free survival of HGSOC patients at this hospital, suggesting that her *RNASEH2B* truncation could have been of beneficial effect.

We aimed to test this hypothesis further using gene expression data from previous ovarian cancer cohorts. In fact, an in silico analysis of publicly available TCGA data using KMplotter provided supportive evidence for an improved progression-free survival in patients with reduced *RNASEH2B* expression (HR 1.53, 95% CI 1.27–1.86, *p* = 9.7 × 10^− 6^; Fig. 1b). The beneficial effect was largely restricted to patients with full success of surgery (HR 1.84, 95% CI 1.31–2.57, *p* = 3.1 × 10^− 4^ with optimal debulking; HR 1.17, 95% CI 0.85–1.60, *p* = 0.34 with suboptimal debulking). When the analysis was restricted to patients with platinum-taxane therapy, there was a significantly better progression-free survival for the low-expression cohort in patients with optimal debulking (HR 1.78, 95% CI 1.21–2.61, *p* = 2.8 × 10^− 3^). Median overall survival also was increased from 38 months in the high-expression cohort to 48 months in the low-expression cohort (HR 1.28, 95% CI 1.00–1.49, *p* = 0.05), and the survival advantage was again restricted to patients with optimal debulking (HR 1.65, 95% CI 1.08–2.51, *p* = 0.02). A similar result was obtained from the analysis of 373 ovarian cancers listed in The Human Protein Atlas [26] where patients with low *RNASEH2B* levels tended to have a longer survival than patients with high *RNASEH2B* levels (5-year-survival 33 months vs. 28 months, *p* = 0.01). When testing the other RNAse H2 genes with KMplotter, a decreased level of *RNASEH2A* showed no benefit (HR 0.86, 95% CI 0.78–0.98, p = 0.02 for all patients; HR 0.84, 95% CI 0.69–1.02 for patients with optimal debulking *p* = 0.07), whereas the analysis of *RNASEH2C* yielded results similar to *RNASEH2B*, indicating an improved progression-free survival in patients with reduced *RNASEH2C* levels (HR 1.55, 95% CI 1.28–1.87, *p* = 5.4 × 10^− 6^ for all patients; HR 1.80, 95% CI 1.30–2.89, *p* = 3.2 × 10^− 4^ for patients with optimal debulking).

## Discussion

Faulty rNTP insertions into the human genome are a common event during DNA replication, estimated to occur at about 1 in 6500 nucleotides [17]. As they have detrimental impact on DNA structure and on replication fork progression, they need to be removed by RNase H1 (for stretches of ribonucleotides) or RNase H2 (for single ribonucleotides) [14]. Human RNase H2 shows strong conservation and comprises RNASEH2A, the catalytic subunit, as well as RNASEH2B and RNASEH2C [4, 14]. Loss of any of its subunits renders the enzyme complex inactive [12, 14]. This results in low level of DNA damage which probably explains the association of RNase H variants with inflammatory disorders but also might be expected to support cancer development. However, the role of RNase H2 in hereditary cancer has been difficult to assess because most patients with Aicardi-Goutières syndrome and pathogenic RNase H2 germline variants have a short lifespan [5]. Nevertheless, it has been proposed that genome instability through RNase H2 impairment upon p53 loss can lead to oncogenic rearrangements and cancer development [14]. Specifically *RNASEH2B* deletions were reported in 57% of chronic lymphocytic leukemias and 36% of aggressive prostate cancers [28]. Interestingly, an exome sequencing study of 491 cases has proposed *RNASEH2B* as a candidate gene for prostate cancer [20]. A possible role for ovarian cancer had not been investigated. Data mining via the TumorPortal indicates four truncating or splice variants of *RNASEH2B* in epithelial cancers (2 in lung adenocarcinoma, 1 in melanoma and 1 in endometrial carcinoma) but none among 316 ovarian cancers.

In the present study, we have aimed to systematically assess the mutational spectrum of the RNase H2 subunit genes *RNASEH2A, RNASEH2B* and *RNASEH2C* in a relatively large case-control series of 602 German patients with EOC and 940 healthy German females to elucidate their possible contribution to ovarian cancer risk and prognosis. Only one patient was found to harbor a bona fide loss-of-function variant, p.C44X in *RNASEH2B*. This variant is listed in gnomAD with a single heterozygote out of 125,644 individuals sequenced, confirming that it is very rare in the general population. Further inspection of our patient revealed a particularly long survival after platinum-based therapy despite some unfavorable prognostic parameters such as high grade serous histology and nodal-positive status. It is possible that the *RNASEH2B* truncating variant modifies survival in such patients. Given the importance of HDR under conditions of rNMP accumulation and PARP1 trapping [28], RNase H2 impairment may assist chemotherapy efficiency when HDR mechanisms are overloaded. Recent evidence indicates that RNase H2 deficiency overall inhibits the exonucleolytic resection of DNA break ends [6], and the same may happen in single-stranded gap filling during or after DNA replication [1, 27]. In line with the latter, *RNASEH2B* is also synthetically lethal with ATR inhibition [27]. Alternatively, RNase H2 deficiency may amplify the mutagenic load in an indirect manner, such as by decreasing the efficiency of MMR [9, 14, 17]. It is also possible that a cytotoxic T-cell response is activated by the absence of RNase H2 which may contribute to the suppression of tumor progression. Such a mechanism has been suggested for *RNASEH2C* as a metastasis susceptibility gene in breast cancer [7].

Consistent with a prognostic role of RNAse H2 in ovarian cancer, our mining of the TCGA database revealed a markedly improved progression-free survival in ovarian cancer patients with reduced *RNASEH2B* expression at a significance level of *p* < 10^− 5^. A similar result was obtained for *RNASEH2C*, which encodes the other regulatory subunit, though not for *RNASEH2A* encoding the catalytic core unit. The prognostic impact of *RNASE2B* and *RNASE2C* levels was maintained significant in the subset of patients with optimal debulking and platinum/taxane-based therapy. If confirmed by other studies, *RNASEH2B* and *RNASEH2C* could emerge as promising targets to improve the outcome of this standard treatment.

In summary, we have shown that pathogenic germline variants in the genes encoding the RNase H2 complex are uncommon in German ovarian cancer patients. We report one patient with a truncating variant in *RNASEH2B* whose longer survival appears consistent with the association of *RNASEH2B* mRNA levels and prolonged survival in previously published ovarian cancer cohorts. Further studies will be required to elucidate how RNase H2 function and regulation affects the prognosis of patients with different ovarian cancer subtypes and treatment regimens.

## Supplementary Information

**Additional file 1: Supplementary Figure 1:** Identified missense and truncating variants of RHASEH2B.

**Additional file 2: Supplementary Table S1:** Primer sequences for *RNASEH2A*, *RNASEH2B* and *RNASEH2C* amplification.

## Data Availability

The datasets used and/or analysed during the current study are available from the corresponding author on reasonable request.
