# Supplementary material for: Germline variation of Ribonuclease H2 genes in ovarian cancer patients
Source: J Ovarian Res. 2020 Dec 22;13:146. doi: 10.1186/s13048-020-00753-1 (PMC7756920; doi:10.1186/s13048-020-00753-1)
Supplement: Supplementary file 1 — Additional file 1: Supplementary Figure 1: Identified missense and truncating variants of RHASEH2B. [file 13048_2020_753_MOESM1_ESM.docx]

**Supplementary Figure 1:** Identified missense and truncating variants of RHASEH2B


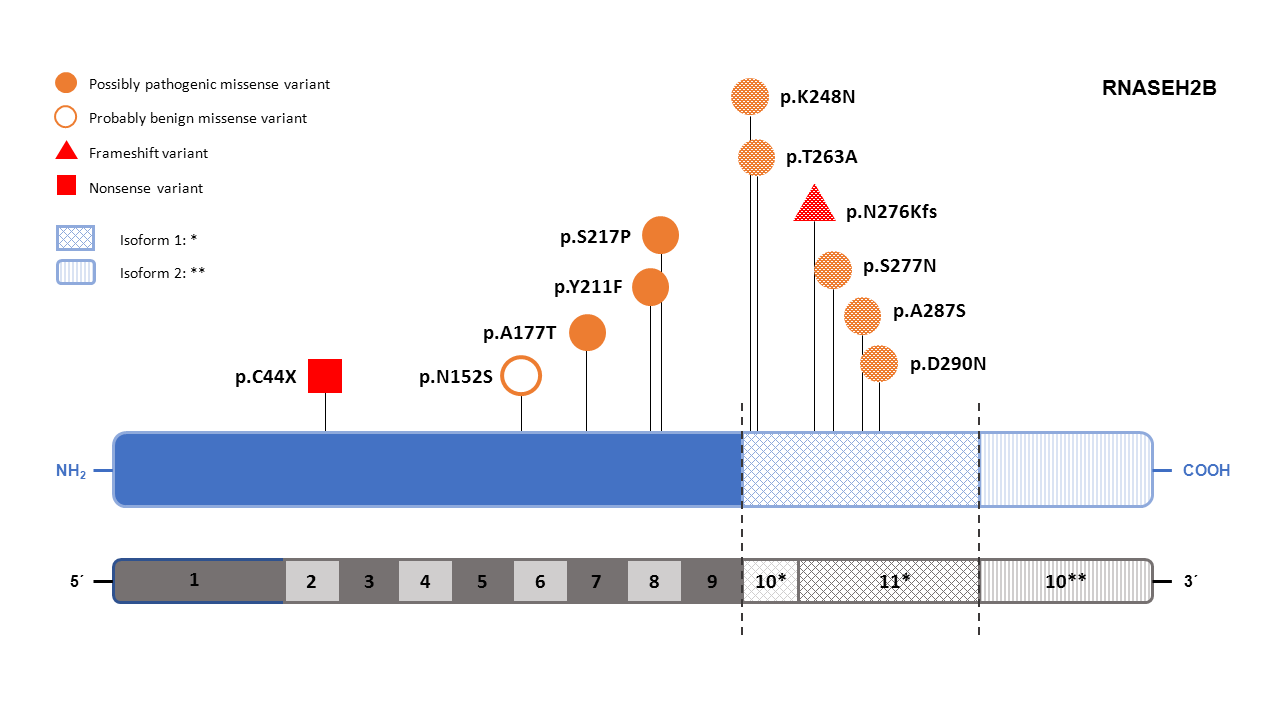


Legend to Supplementary Figure S1:

Distribution of identified missense and truncating variants across the coding sequence of *RHASEH2B*. Symbols distinguish variant types as indicated. Hatched areas indicate alternatively spliced exons. The exons 10* and 11* are used in *RNASEH2B* transcript NM_024570.4, whereas they are skipped and the exon 10** is used in *RNASEH2B* transcript NM_001142279.2. Figure not drawn to scale.
