# Supplementary material for: Germline variation of Ribonuclease H2 genes in ovarian cancer patients
Source: J Ovarian Res. 2020 Dec 22;13:146. doi: 10.1186/s13048-020-00753-1 (PMC7756920; doi:10.1186/s13048-020-00753-1)
Supplement: Supplementary file 2 — Additional file 2: Supplementary Table S1: Primer sequences for RNASEH2A, RNASEH2B and RNASEH2C amplification. [file 13048_2020_753_MOESM2_ESM.docx]

**Supplementary Table S1:** Primer sequences for *RNASEH2A*, *RNASEH2B* and *RNASEH2C* amplification

| **Assay Name** | **Forward Primer** | **Reverse Primer** | **Size(bp)** | **Chr** | **From** | **To** |
| --- | --- | --- | --- | --- | --- | --- |
|  |  |  |  |  |  |  |
| RNASEH2A_1 | TGTTCTGCCCGCGGATTG | GTTTCCCGCATCCTCCGTA | 298 | chr19 | 12806557 | 12806854 |
| RNASEH2A_2 | AAGCAGTGATGATAGAACAGGGA | CTTTGAGTCTGGGAGGAGGTTTG | 299 | chr19 | 12806915 | 12807213 |
| RNASEH2A_3 | CAGGTGAGAACTGAAAGGGGAG | GGAACAGAGCCCAGTTAATCTCA | 279 | chr19 | 12807131 | 12807409 |
| RNASEH2A_4 | CTGTTTGCGAAAATGGAGGACAC | TGACCAAATGGGGACAACTTACA | 299 | chr19 | 12807241 | 12807539 |
| RNASEH2A_5 | AGGCTAGAGCATTGGTACAGTTG | CTGGAATCTCCCTCCTTCCCTTT | 285 | chr19 | 12810013 | 12810297 |
| RNASEH2A_6 | CAAAGCAGATGCCCTCTACCC | AGCCCTGGCCATTTCAACATTAG | 295 | chr19 | 12810157 | 12810451 |
| RNASEH2A_7 | GCTTGAATGTTATGGTGCAACT | ATGACAGTGACCACTTACACAGG | 282 | chr19 | 12813030 | 12813311 |
| RNASEH2A_8 | GTTGCTCCCTGTGTAAGTGGTC | GTCCCAAAAGTACATCCCCTACG | 275 | chr19 | 12813282 | 12813556 |
|  |  |  |  |  |  |  |
| RNASEH2B_1 | TTGTCCGGGCACACAC | GGGACCGAATTTCGTTTCGTTT | 296 | chr13 | 50909642 | 50909937 |
| RNASEH2B_2 | CGTAACACGAGCAGCAG | TCCGCCAGTTCTTGGG | 300 | chr13 | 50909888 | 50910187 |
| RNASEH2B_3 | GGGTAAAGTAAGGTGAGCAACAA | TTCTTCTCCTCACGGTGCCAT | 270 | chr13 | 50927339 | 50927608 |
| RNASEH2B_4 | AGGAACATTCGTCAGAGATACTGG | CCACGTGTGAAGAATGGAAAAGC | 277 | chr13 | 50929375 | 50929651 |
| RNASEH2B_5 | AGACTCTGAGGCCTGTATAAGAA | AGGAGAGAGAGGAGATCAAACAA | 298 | chr13 | 50930536 | 50930833 |
| RNASEH2B_6 | GGAATAGGTGGCTTCTGCACTAA | GCAAGGTCCATCCTTTCTACCAG | 286 | chr13 | 50934763 | 50935048 |
| RNASEH2B_7 | ACATTTCAAGTATGTTCTCAGGTTTG | TACCACAAGGCACTAGAGAGGAA | 255 | chr13 | 50943169 | 50943423 |
| RNASEH2B_8 | AGTTAAGTTGAAAATACCCTGCCTT | CACACCTGCCACAATGTGTATGA | 295 | chr13 | 50945380 | 50945674 |
| RNASEH2B_9 | AGGCTAGAGCTTAAGTGAGTTCC | CTGGGGAAACCAAGAAGCAAATG | 270 | chr13 | 50947857 | 50948126 |
| RNASEH2B_10 | AAGTTGGCCCTGTCTTTCTGTTT | GTGATGGAGAGCCCAGTACAATC | 275 | chr13 | 50949390 | 50949664 |
| RNASEH2B_11 | AAAAAGTAAGCCCTTTGCTGGGT | CATCACGCACTGAGTATGAACGA | 270 | chr13 | 50953766 | 50954035 |
| RNASEH2B_12 | ACATGCAGTCTTCTTTGATTTCCA | TCCCCCAACCAAAGGTAGTCATT | 291 | chr13 | 50956276 | 50956566 |
| RNASEH2B_13 | TGTCTGAGGTCATCTCTTAGAAGC | GCAGAAGAAAAAGTCCGAGTCCT | 272 | chr13 | 50969773 | 50970044 |
|  |  |  |  |  |  |  |
| RNASEH2C_1 | GCTCCTTTATTGGGGTGATGGAA | TTCTACTGGGTAGTGTGTGGGAA | 272 | chr11 | 65719695 | 65719966 |
| RNASEH2C_2 | ATTGTGCTCCCCAGCCCAT | GACTGACGACCAAGAGGAGGAG | 293 | chr11 | 65719989 | 65720281 |
| RNASEH2C_3 | GGCTCAGCATCGGGACTAC | TCCTGCCTGAGGCCCTTT | 272 | chr11 | 65720180 | 65720451 |
| RNASEH2C_4 | CAGTGTGGCGGGTACGG | GGGGAAGGAAGGAAGGAAAAAA | 300 | chr11 | 65720672 | 65720971 |
